# Catalytic and Photocatalytic Nitrate Reduction Over Pd-Cu Loaded Over Hybrid Materials of Multi-Walled Carbon Nanotubes and TiO_2_

**DOI:** 10.3389/fchem.2018.00632

**Published:** 2018-12-20

**Authors:** Cláudia G. Silva, Manuel F. R. Pereira, José J. M. Órfão, Joaquim L. Faria, Olívia S. G. P. Soares

**Affiliations:** Laboratory of Separation and Reaction Engineering-Laboratory of Catalysis and Materials (LSRE-LCM), Faculdade de Engenharia, Universidade do Porto, Porto, Portugal

**Keywords:** photocatalysis, catalytic reduction, nitrate, titanium dioxide, carbon nanotubes, palladium, copper

## Abstract

TiO_2_ and carbon nanotube-TiO_2_ hybrid materials synthesized by sol-gel and loaded with 1%Pd−1%Cu (%*wt*.) were tested in the catalytic and photocatalytic reduction of nitrate in water in the presence of CO_2_ (buffer) and H_2_ (reducing agent). Characterization of the catalysts was performed by UV-Vis and fluorescence spectroscopy, X-ray diffraction, temperature programed reduction, N_2_ adsorption, and electron microscopy. The presence of light produced a positive effect in the kinetics of nitrate removal. Higher selectivity toward nitrogen formation was observed under dark condition, while the photo-activated reactions showed higher selectivity for the production of ammonium. The hybrid catalyst containing 20 %wt. of carbon nanotubes shows the best compromise between activity and selectivity. A mechanism for the photocatalytic abatement of nitrate in water in the presence of the hybrid materials was proposed, based in the action of carbon nanotubes as light harvesters, dispersing media for TiO_2_ particles and as charge carrier facilitators.

## Introduction

Nitrate is a naturally occurring ion that is part of the nitrogen cycle, in which nitrogen species are switched between organisms and the environment. The increasing use of inorganic nitrogenous fertilizers, the disposal of wastes (mainly from oxidation of nitrogenous waste products in human and animal excreta) and changes in land use are the main causes accounting for the progressively increasing levels of this pollutant in groundwater supplies. Nitrate ion (${{\text{NO}}_{3}}^{-}$) is the stable form of combined nitrogen for oxygenated systems and is potentially hazardous for humans, since it can be transformed into nitrite in the human body, which may cause the blue baby syndrome, being also a precursor of carcinogenic nitrosamines (Kapoor and Viraraghavan, 1997). Moreover, it may cause the eutrophication of rivers and lakes. The maximum contaminant level in drinking water of nitrogen species as nitrate, nitrite, and ammonium is 50, 0.5, and 0.5 mg/L, respectively (EU Water Framework Directive^1^).

The removal of nitrate from water constitutes a great challenge to safeguarding drinking water resources of suitable quality. In this context, a great effort has been put in the development of water technologies capable to address the environmental and health concerns. Conventional methods are based in physical-chemical treatment processes (reverse osmosis, ion exchange, and electrodialysis) and biological denitrification.

Heterogeneous catalytic systems like catalytic and photocatalytic reduction of nitrate have been shown to be promising processes compared to conventional treatments (Sá et al., 2009; Soares et al., 2011a, 2014; Luiz et al., 2012; Shand and Anderson, 2013). Catalytic nitrate reduction occurs by consecutive and parallel reactions where nitrate is reduced to nitrite, which is converted to ammonium, as undesired by-product, and to nitrogen, as desired product. The main issue of this process is the selectivity toward nitrogen, which is often compromised. Bimetallic catalysts composed by a noble metal (Pd, Pt, or Rh) and a promoter metal (Cu, Sn, or In) supported on different materials are the most used for catalytic hydrogenation of nitrate (Soares et al., 2009; Calvo et al., 2010; Marchesini et al., 2010; Wada et al., 2012), although monometallic catalysts also present some activity depending on the support (Barrabés et al., 2010; Anderson, 2011; Devadas et al., 2011). Pd-Cu catalysts are normally the most efficient, Pd-Sn and Pd-In also presenting good performances (Martínez et al., 2017). In the case of photocatalytic reduction, besides the type of metal catalysts, several other conditions such as the catalyst support, the pH of the solution, the irradiation source, nature of the reducing agent or electron donor (hole scavenger) also affect the performance of the process. H_2_ and CO_2_ are usually used in catalytic reduction reactions, in which H_2_ serves as reductant and CO_2_ as buffer (Prusse et al., 2000). In the case of the photo-assisted catalytic process, oxalic acid, formic acid, or methanol are the most used hole scavengers (Zhang et al., 2005; Doudrick et al., 2013).

Titanium dioxide-based materials are the most widely used catalysts for the photocatalytic reduction of nitrate, the bimetallic Pd-Cu/TiO_2_ and Pt-Cu/TiO_2_ being the ones showing most promising results. On the other hand, Pd-Cu catalysts supported on carbon materials and also in metal oxides are the most used for nitrate catalytic reduction. TiO_2_-based catalysts are normally highly active for nitrate removal, yet, due to their capacity to drive hydrogenation, low nitrogen selectivity is reached (Sá et al., 2005). It has been reported that the activity of Pd-Cu bimetallic catalysts supported on carbon materials was higher than the same Pd-Cu catalyst supported on a metal oxide, such as TiO_2_, Al_2_O_3_, SiO_2_, or ZrO_2_, at the same operating conditions, due to their surface chemistry and higher metals dispersion (Sakamoto et al., 2006; Soares et al., 2011a). Moreover, the coupling of carbon nanotubes with TiO_2_ has proved to induce a positive effect in the activity and selectivity of Pd-Cu catalysts for the catalytic reduction of nitrate into N_2_ (Soares et al., 2011a).

Following the previous findings, in the present work we explored the synergies between carbon nanotubes (CNT) and titanium dioxide in both catalytic and photocatalytic reduction of nitrate in water. For that purpose, hybrid materials of TiO_2_ with different CNT contents, and loaded with 1%Pd and 1%Cu (wt.%) were evaluated as catalysts. Hydrogen and carbon dioxide were used as reducing and pH buffer agents, respectively. The materials were tested under the same experimental conditions, the only difference between the two processes being the introduction of a near-UV to visible light source in the case of the photocatalytic reactions.

## Experimental

### Catalysts Preparation

Multi-walled carbon nanotubes synthesized by catalytic decomposition of CH_4_ were purchased from Shenzhen Nanoport Co. Ltd (purity > 95%, diameter < 10 nm; length = 5–15 μm). TiO_2_ and CNT–TiO_2_ composite catalysts were prepared through an acid-catalyzed sol–gel procedure, as described elsewhere (Silva and Faria, 2010). Briefly, TiO_2_ was prepared by dissolving Ti(OC_3_H_7_)_4_ (Aldrich 97%) in ethanol. The solution was magnetically stirred for 30 min, and then nitric acid (Fluka 65%) was added.

For the composite catalysts preparation, a certain amount of CNT was added to the Ti(OC_3_H_7_)_4_ ethanol solution. The mixture was kept stirring until a homogenous gel was formed. The gel was left aging in air for 5 days. The resulting material was then crushed into a fine powder (particle size < 100 μm). The powders were calcined at 400°C under a flow of N_2_ for 2 h to obtain TiO_2_ or CNT–TiO_2_ hybrid materials. Catalysts were labeled as XCNT–TiO_2_, where X (5, 10, 20, 50, 70, and 90) corresponds to the weight percentage of CNT in the material.

The monometallic (Pd) and bimetallic (Pd-Cu) catalysts were prepared by incipient wetness impregnation and co-impregnation, respectively. Briefly, aqueous solutions containing the proper mass of the corresponding salts [PdCl_2_, Alfa Aesar 99.9%; Cu(NO_3_)_2_, Riedel-de Haen 99%] were added dropwise to TiO_2_ and CNT-TiO_2_ materials. In the case of the bimetallic catalyst, the materials were co-impregnated with a solution containing both precursor salts. The palladium and copper contents were fixed at 1%Pd-1%Cu and 1%Pd (weight percentages). After impregnation, the metal-loaded materials were dried in an oven at 100°C for 24 h. Then, the catalysts were heat treated under a nitrogen flow at 200°C for 1 h. At the end of this period, the gas stream was switched to hydrogen for 3 h to promote metals reduction. Finally, the materials were left to cool down to room temperature under a nitrogen flow.

### Catalysts Characterization

Powder X-ray Diffraction (XRD) analysis was performed on a Philips X'PertMPD diffractometer (Cu-Ka = 0.15406 nm). The Brunauer-Emmett-Teller (BET) specific surface area (S_BET_) was determined from N_2_ adsorption-desorption isotherms at 196°C, in a Quantachrome Nova 4200e apparatus. Temperature programmed reduction (TPR) was carried out in an AMI-200 (Altamira Instruments) system. The H_2_ consumption was followed by a thermal conductivity detector (TCD) and by a mass spectrometer (Dymaxion 200 amu, Ametek). Transmission electron microscopy (TEM) micrographs were obtained using a LEO 906E microscope operating with an accelerating voltage of 120 kV. Diffuse reflectance (DR) UV-Vis spectra of the powder samples were recorded on a JASCO V-560 UV-Vis spectrophotometer, equipped with an integrating sphere attachment (JASCO ISV-469). The reflectance spectra were converted by the instrument software (JASCO) to equivalent absorption Kubelka–Munk units. Steady-state photoluminescence (PL) spectra were recorded at room temperature on a JASCO FP-8300 spectrofluorometer equipped with a 150 W Xe lamp. The morphology and elemental mapping of the materials was obtained by SEM/EDXS analysis using a FEI Quanta 400FEG ESEM/EDAX Genesis X4M instrument.

### Catalytic and Photocatalytic Nitrate Reduction Experiments

The catalytic and photocatalytic experiments were carried out in a glass cylindrical reactor. Initially, 190 mL of deionised water and 100 mg of catalyst were fed into the reactor. When used, a gas mixture of H_2_ and CO_2_ [1:1 flow rate = 200 cm^3^ (STP) min^−1^] was passed through the reactor to remove the dissolved oxygen; CO_2_ acts as pH buffer (pH = 5.5). A Heraeus TQ 150 medium pressure mercury vapor lamp (λ_exc_ = 254, 313, 365, 436, and 546 nm) was used as radiation source. The lamp was located axially in the reactor and held in a quartz immersion tube. A DURAN® glass jacket was used as water circulating cooling system (temperature maintained at 25°C) and as a filter for cutting-off low wavelength UV lines and letting pass radiation in the near-UV to visible light range (λ_exc_ ≥ 365 nm). Before turning illumination on, the solution was magnetically stirred in the dark for 15 min. After that period, 10 mL of a nitrate solution, prepared from NaNO_3_ (Sigma-Aldrich 99%), was added to the reactor, in order to obtain an initial ${{\text{NO}}_{3}}^{-}$ concentration of 100 mg L^−1^. The first sample was taken out just before the light was turned on, in order to determine the initial nitrate concentration in solution. The catalytic (dark) experiments were carried out under the same experimental conditions, but in the absence of light.

Samples were withdrawn regularly from the reactor, and centrifuged before determination of ${{\text{NO}}_{3}}^{-}$, ${{\text{NO}}_{2}}^{-}$, and ${\text{NH}}_{4}{}^{+}$ concentrations. ${{\text{NO}}_{3}}^{-}$ and ${{\text{NO}}_{2}}^{-}$ were simultaneously determined by HPLC using a Hitachi Elite Lachrom system equipped with a diode array detector. The stationary phase was a Hamilton PRP-X100 column (150 × 4.1 mm) working at room temperature, under isocratic conditions. The mobile phase was a solution of 0.1 M NaCl:CH_3_OH (45:55). The concentration of ${\text{NH}}_{4}{}^{+}$ was determined by potentiometry.

Palladium and copper leaching was assessed after each experiment by atomic absorption spectrometry (UNICAM 939/959), the absence of metals (within the experimental error) being confirmed for all the cases.

Reproducibility tests were performed for selected experiments, the results being in agreement with a maximum error of about 2.5%. ${{\text{NO}}_{3}}^{-}$ conversion and the selectivity to ${{\text{NO}}_{2}}^{-}$ and ${\text{NH}}_{4}{}^{+}$ were calculated as described elsewhere (Soares et al., 2014).

## Results and Discussion

### Catalysts Characterization

**Figure 1:**
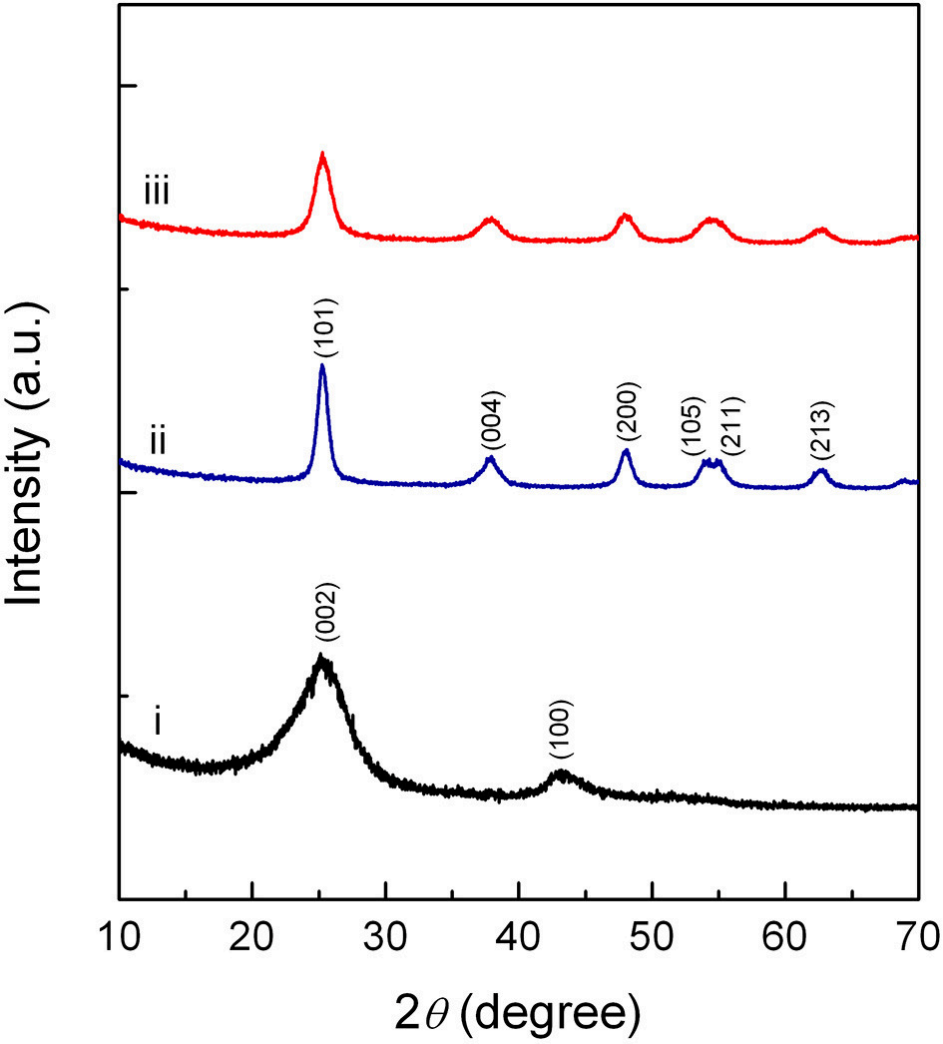
X-ray diffraction patterns of neat CNT (i), Pd-Cu/TiO_2_ (ii), and Pd-Cu/20CNT-TiO_2_ (iii).

**Figure 2:**
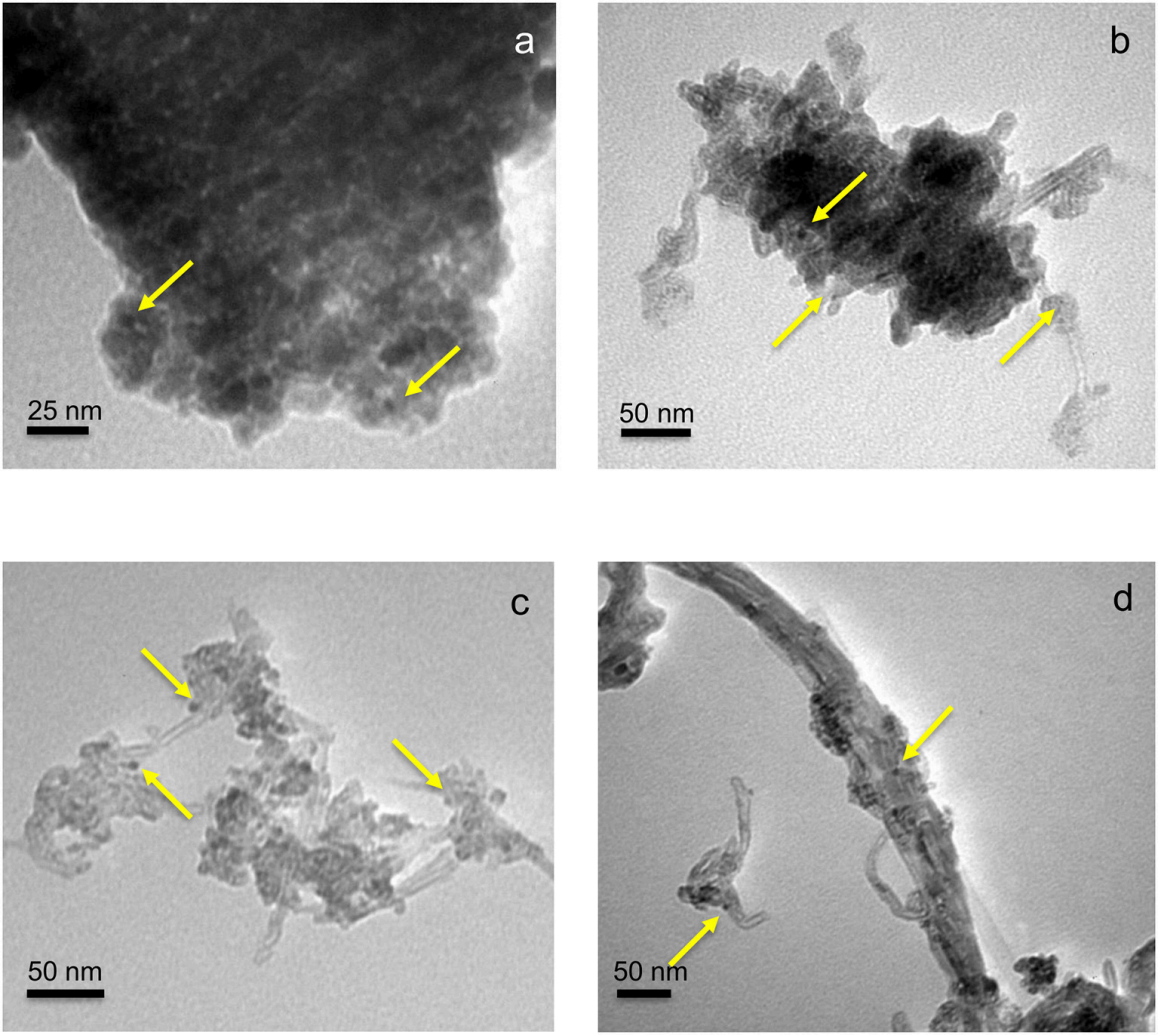
TEM micrographs of TiO_2_ **(a)**, 5CNT-TiO_2_ **(b)**, 20CNT-TiO_2_ **(c)**, and 70CNT-TiO_2_ **(d)** loaded with 1% Cu and 1% Pd (*wt*. %). The arrows indicate the presence of metal nanoparticles.

XRD patterns of neat CNT, Pd-Cu/TiO_2_, and Pd-Cu/20CNT-TiO_2_ are displayed in Figure 1. Typical (002) and (100) diffraction lines are evident in the XRD pattern of CNT. XRD analysis of TiO_2_ and CNT-TiO_2_ composites revealed that only anatase phase is present in neat TiO_2_ and composite catalysts. The XRD patterns of the Pd-Cu loaded CNT–TiO_2_ materials are very similar to the one of Pd-Cu/TiO_2_, with the CNT contribution hardly been identified. The Cu and Pd phases were not detected by XRD, which must be related to the low metal percentages (1%wt.). Anatase crystallites of 8.5 nm average size were found for neat TiO_2_, as determined by the Scherrer equation and confirmed by TEM (Figure 2a). The sizes of the anatase crystallites present at the composite catalysts decreased with increasing carbon content, suggesting that CNT may act as dispersing medium for TiO_2_ particle precursors during the crystallization process (Table 1).

**Table 1:** Surface area, carbon content, and dimensions of the anatase crystallites of TiO_2_ and CNT-TiO_2_ composites.

**Catalyst**	**S_BET_** ** (m^2^ g^−1^)**	**S_BET, calc_** ** (m^2^ g^−1^)**	**C_TG_** ** (%)**	**d_A_** ** (nm)**
TiO_2_-SG	107	–	–	8.5
CNT	185	–	–	-
5CNT-TiO_2_	70	110	3.5	11.2
10CNT-TiO_2_	94	113	7.6	9.4
20CNT-TiO_2_	131	120	17	8.3
50CNT-TiO_2_	147	143	46	7.2
70CNT-TiO_2_	111	130	71	6.5*^a^*
90CNT-TiO_2_	104	174	86	*n.d*.

a *Determined by TEM; n.d., not determined*.

The BET surface areas (S_BET_) of TiO_2_ and CNT-TiO_2_ materials are listed in Table 1. Materials with lower carbon content, namely 5CNT–TiO_2_ and 10CNT–TiO_2_ composites, showed surface areas lower than the ones estimated through the mass composition of the composites (S_BET, calc_) and even lower than for neat TiO_2_. These results indicate that the presence of low amounts of CNT induces the formation of big TiO_2_ crystallite agglomerates, therefore decreasing the surface area of the composite catalyst (Figure 2b).

With the carbon content increasing up to 20% (20CNT–TiO_2_), the presence of a larger amount of CNT seems to prevent TiO_2_ particles from agglomerating, thus increasing the surface area, which was even higher than the calculated (S_BET, calc_). This was confirmed by TEM (Figure 2c), where TiO_2_ particles of very small dimensions can be observed surrounding the sidewalls of CNT. In the case of the composite with similar amounts of TiO_2_ and CNT phases (50CNT-TiO_2_), the values for S_BET_ and S_BET, calc_ were similar. Nevertheless, a further increase on the amount of CNT revealed to have a detrimental effect on the surface area of the resulting composites. A decrease in the S_BET_ in relation to the calculated values of 15 and 40% was observed for 70CNT-TiO_2_ and 90CNT-TiO_2_ materials, respectively, which may be attributed to the formation of CNT bundles decreasing the accessible surface area, as could be visualized by TEM for 70CNT-TiO_2_ (Figure 2d). Due to the low amount of metals used, the textural properties of the metal-loaded catalysts remained practically unchanged compared to the pristine supports. The presence of metal particles of very small dimensions (lower than 10 nm) loaded on the TiO_2_-based materials could be observed by TEM (Figure 2).

**Figure 3:**
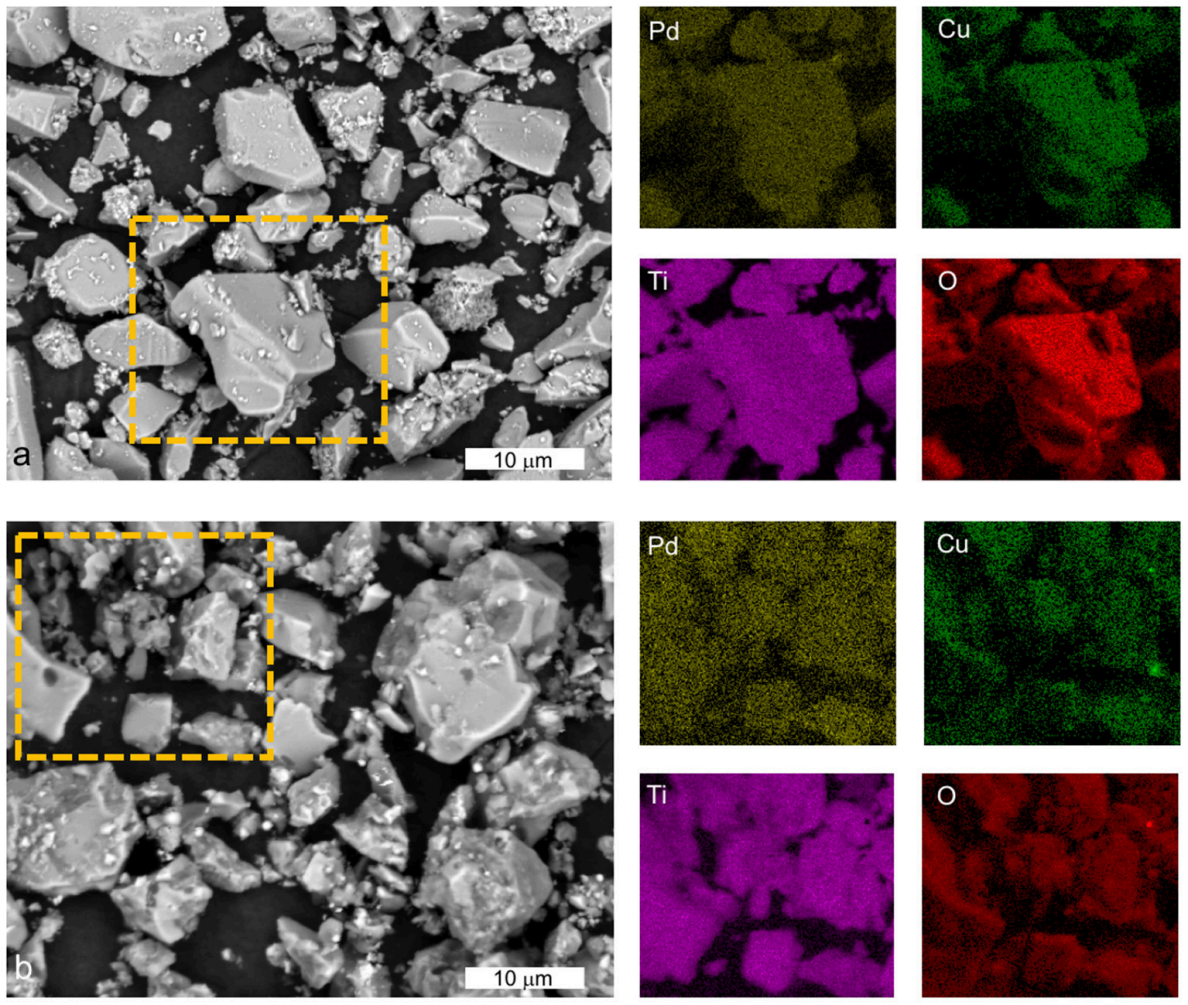
SEM-EDX analysis of Pd-Cu/TiO_2_ **(a)** and Pd-Cu/20CNT-TiO_2_ **(b)** with the respective elemental mapping for Pd, Cu, Ti, and O in the selected regions (dashed rectangle).

SEM-EDXS analysis of Pd-Cu/TiO_2_ (Figure 3a) and Pd-Cu/20CNT-TiO_2_ (Figure 3b) revealed that Pd and Cu nanoparticles are well dispersed in the catalysts, with no apparent prevalence of one of the metals.

The carbon content of the CNT-TiO_2_ composites determined by TG analysis (C_TG_) agrees fairly well with the nominal percentage, indicating negligible gasification of CNT during the calcination step (Table 1).

**Figure 4:**
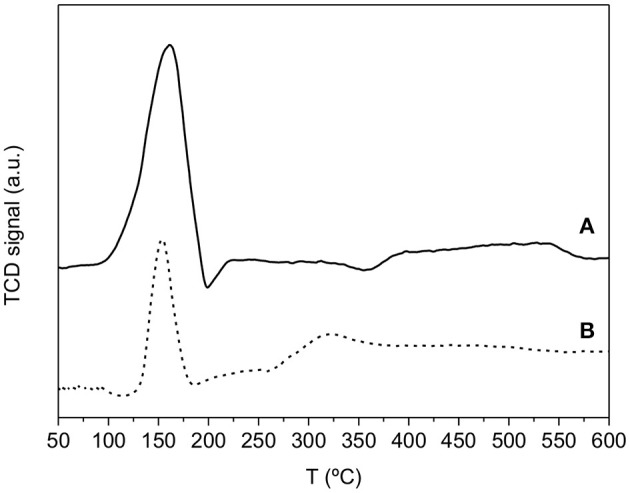
TPR profiles of Pd-Cu loaded on TiO_2_ **(A)** and on 20CNT-TiO_2_ **(B)**.

Figure 4 shows the TPR profiles for both TiO_2_ and 20CNT-TiO_2_ materials, which were obtained before heat-treating the metal salt-loaded supports. Both materials show a reduction peak centered at 150°C, assigned to the reduction of Cu oxides promoted by the presence of Pd (Soares et al., 2011a). The thermal treatment under H_2_ should produce Pd and Cu particles in the reduced form, i.e., Pd^0^ and Cu^0^ (Soares et al., 2010, 2011a). Similar results were obtained for the remaining CNT-TiO_2_ composite materials.

**Figure 5:**
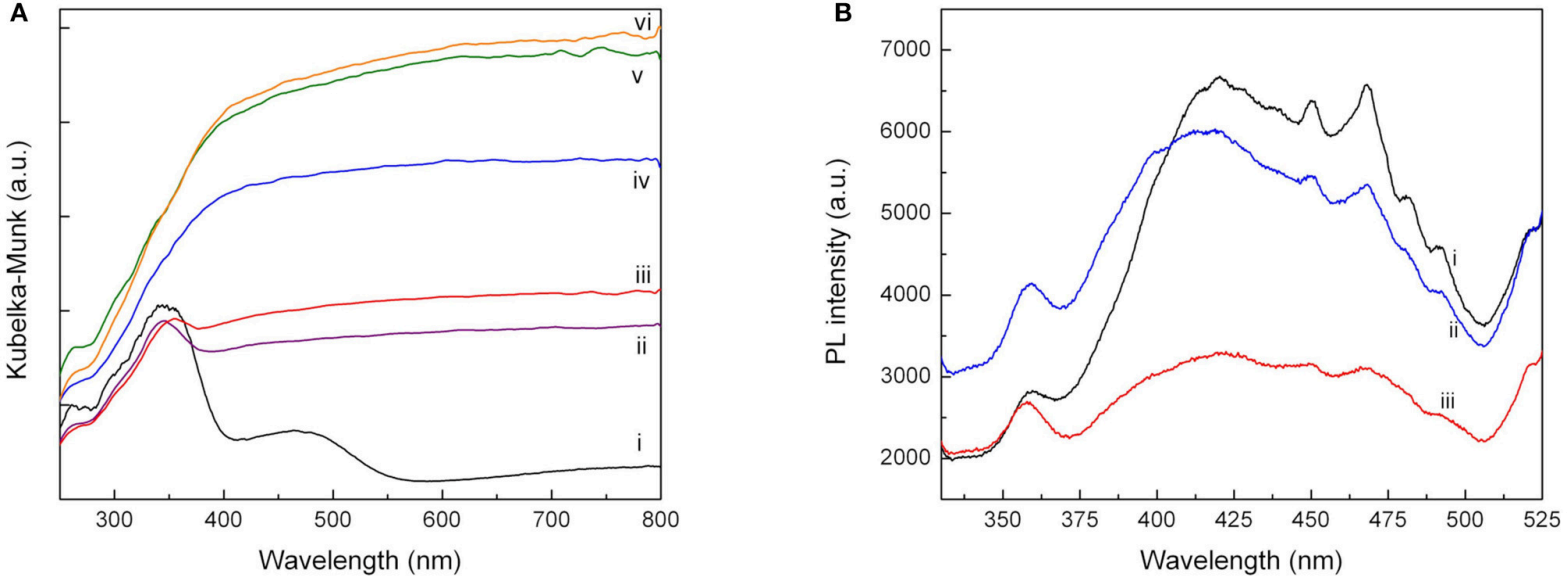
**(A)** Diffuse reflectance UV-Vis spectra of Pd-Cu-loaded TiO_2_ (i); 5CNT-TiO_2_ (ii); 10CNT-TiO_2_ (iii); 20CNT-TiO_2_ (iv); 50CNT-TiO_2_ (v); CNT (vi); **(B)** PL spectra of neat TiO_2_ (i), Pd-Cu/TiO_2_ (ii) and Pd-Cu/20CN-TiO_2_ (iii).

DR UV-Vis analysis of Pd-Cu/TiO_2_ (Figure 5A) shows the TiO_2_ characteristic absorption band at wavelength below 400 nm, a band peaking at c.a. 480 nm attributed to the presence of Pd and a broad absorption band rising from 550 nm due to the occurrence of Cu species (López et al., 2009; Wu et al., 2009; Soares et al., 2014).

As expected, the presence of CNT led to a rise of light absorption in the visible spectral region, increasing with the CNT content on the composite catalysts up to a CNT load of 50 wt.%. This behavior has been attributed not only to the capacity of CNT to absorb visible light but also to an increment of surface electronic species availability and mobility in the composite catalysts due to the introduction of CNT, as already reported in previous studies (Silva and Faria, 2010; Dai et al., 2014). A further increase in the CNT content did not produce any effect on the optical absorption of the composite materials. Moreover, the absorption peaks of the metal species could not be identified in the UV-Vis spectra of the composite materials, which may be attributed to a higher dispersion of the metal particles when supported on the composite materials.

The photoluminescence (PL) spectra were performed for having an insight in the behavior of light-generated electronic species in photocatalysts, since PL emission results from the recombination of electrons and holes. The PL emission spectra of the pure neat TiO_2_ and Pd-Cu loaded TiO_2_ and 20CNT-TiO_2_ materials excited at 280 nm are shown in Figure 5B. The PL signal observed for neat TiO_2_ can be attributed to the transition of electrons from the oxygen vacancies to TiO_2_ valence band (Tahir et al., 2017). After loading TiO_2_ with Pd and Cu, an increase in the PL intensity was observed in the range from 330 to 400 nm, which may be attributed to the higher availability of photoexcited electrons in the bimetallic catalyst. Yet, a decrease in the PL signal intensity in the 400–525 nm range is observed, meaning that electron-hole recombination was decreased by the presence of the metal nanoparticles. Pd-Cu/20CNT-TiO_2_ shows a very significant decrease in the PL intensity as compared with bare and metal loaded TiO_2_, indicating highly efficient inhibition of charge carriers recombination and suggesting the existence of electronic synergies between the metals and the hybrid CNT-TiO_2_ material (Zhang et al., 2012).

### Catalytic and Photocatalytic Nitrate Reduction

TiO_2_ and CNT-TiO_2_ composites loaded with 1%Pd and 1%Cu (wt.%) were used for catalytic and photocatalytic reduction of nitrate in aqueous suspensions. Monometallic Pd-TiO_2_ was used for comparison purposes. H_2_ and CO_2_ were continuously added to the reaction media, acting as reducing agent and pH buffer, respectively. The materials were tested under the same experimental conditions, the only difference between the two processes being the introduction of a near-UV to visible light source in the case of the photocatalytic reactions.

**Figure 6:**
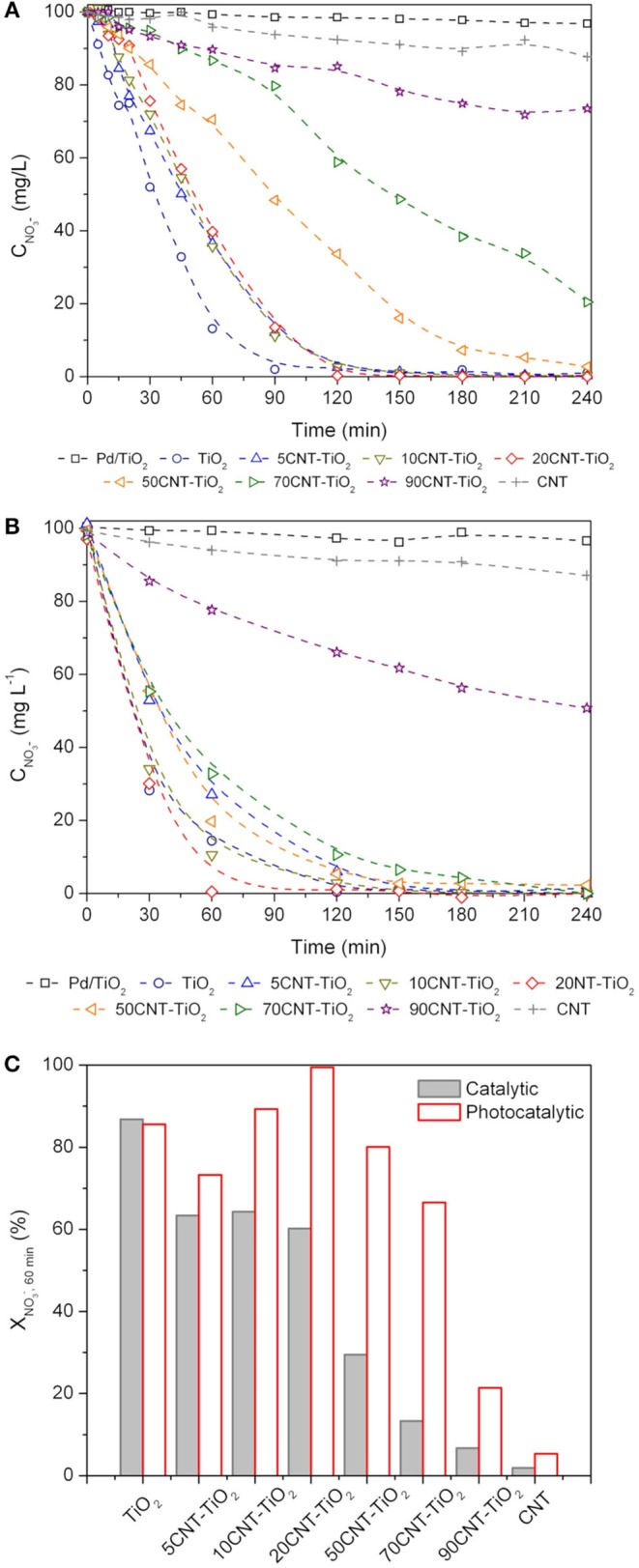
Nitrate concentration (${\text{C}}_{{{\text{NO}}_{3}}^{-}}$) during catalytic **(A)** and photocatalytic **(B)** reactions. Pd/TiO_2_ refers to the monometallic catalyst while all other results refer to Pd-Cu bimetallic catalysts. Nitrate conversion at 60 min (${\text{X}}_{\text{N}{\text{O}}_{3}{}^{-},60\text{ min}}$) using Pd-Cu loaded materials **(C)**.

The performances of the different catalysts for the (dark) catalytic reduction process are presented in Figure 6A. Very low nitrate conversion (4%) was achieved using the monometallic Pd-TiO_2_ catalyst, the presence of Cu being fundamental for the reaction to occur. Moreover, as expected, it can be observed that the support has a crucial influence on the performance of the bimetallic catalysts.

In the case of the catalytic reduction process, Pd-Cu/TiO_2_ was the most efficient catalyst in terms of the kinetics of nitrate removal, with total nitrate conversion being achieved at the end of 90 min of reaction. Similar kinetic behavior was observed for nitrate reduction reactions using the composite materials with a CNT load (Y) of 5, 10, and 20%, leading to total nitrate removal at the end of 120 min of reaction. For higher CNT loads, a progressive detrimental effect in the kinetics of nitrate removal has been observed. Nitrate conversions of 95, 79, and 27% have been obtained at the end of 240 min when using composites with Y = 50, 70, and 90%, respectively. For the Pd-Cu/CNT catalyst only 12% nitrate removal was achieved at the end of the catalytic run.

In general, the presence of light promotes a positive effect in nitrate removal (Figure 6B). As in the case of the (dark) catalytic process, the simultaneous presence of Pd and Cu was a *sine qua non* condition for nitrate conversion to occur. The composite material with the lowest CNT content (Y = 5%) produced a decrease in the efficiency of nitrate abatement compared to Pd-Cu/TiO_2_. Yet, a further increase in the CNT load up to Y = 20% lead to a rise in the rate of nitrate removal. For the composites with higher CNT content (Y = 50, 70, and 90%) a progressive loss in the efficiency toward ${{\text{NO}}_{3}}^{-}$ conversion was observed.

The conversion of nitrate at 60 min of reaction was calculated in order to get a better understanding of the effect of CNT load in the kinetics of ${{\text{NO}}_{3}}^{-}$ removal by both catalytic and photocatalytic routes (Figure 6C). It is notorious that the amount of CNT plays a role in the efficiency of the composite materials. As already mentioned, composite materials underperformed TiO_2_ in catalytic nitrate reduction with a decrease in the nitrate conversion with increasing CNT load. Yet, for the photocatalytic process a positive effect was observed using 10CNT-TiO_2_ and, in particular, 20CNT-TiO_2_ comparing with the experiments using TiO_2_ as support. On the other hand, the bimetallic catalyst supported on 20CNT-TiO_2_ promotes total conversion of nitrate at 60 min of reaction. Reutilization tests were performed using Pd-Cu/20CNT-TiO_2_ and Pd-Cu/TiO_2_ under catalytic and photocatalytic conditions. In both cases the results indicate that the performance of the catalysts was maintained within 5% variation over 3 consecutive runs.

**Figure 7:**
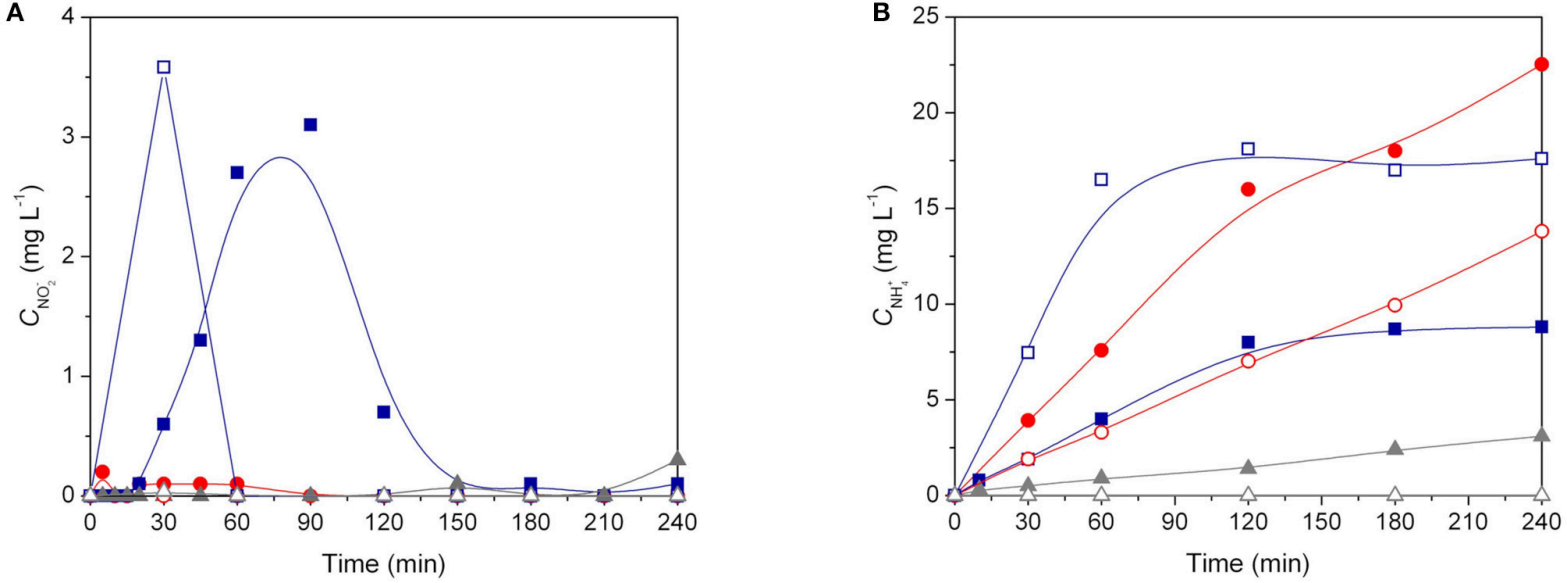
**(A)** ${{\text{NO}}_{2}}^{-}$ and **(B)** ${\text{NH}}_{4}{}^{+}$ concentrations as a function of time during nitrate reduction over Pd–Cu loaded TiO_2_ (● and ○), 20CNT-TiO_2_ (■ and □ ) and CNT (▲ and △) under dark catalytic (solid symbols) and photocatalytic (open symbols) conditions.

Figure 7 shows the nitrite and ammonium profiles during catalytic and photocatalytic reactions using Pd-Cu loaded TiO_2_, CNT, and 20CNT-TiO_2_ catalysts. Residual amounts of ${{\text{NO}}_{2}}^{-}$ and ${\text{NH}}_{4}{}^{+}$ were produced using Pd-Cu/CNT during both catalytic and photocatalytic processes. When Pd-Cu/TiO_2_ was used as catalyst, nitrite is partially transformed into ammonia during the reaction, which is accumulated in the aqueous media. It was found that for the catalytic process, the formation of nitrite and conversion into ammonium is slower than for the photocatalytic process. Also, lower amounts of ammonia were produced in the presence of light, using Pd-Cu/TiO_2_ and Pd-Cu/CNT, meaning a higher selectivity of the photocatalytic process toward N_2_ formation.

For the reactions using Pd-Cu/20CNT-TiO_2_ higher amounts of ${{\text{NO}}_{2}}^{-}$ were found during the catalytic and photocatalytic reactions, which were completely depleted at the end of 180 and 60 min of reaction, respectively (Figure 7A). Yet, contrarious to what was observed for the reactions using Pd-Cu/TiO_2_ and Pd-Cu/CNT, the use of the metal-loaded 20CNT-TiO_2_ catalyst under irradiation lead to the formation of higher amounts of ammonia when compared to the catalytic process (Figure 7B).

### Catalytic and Photocatalytic Nitrate Reduction Mechanisms Using CNT-TiO_2_ Catalysts

**Figure 8:**
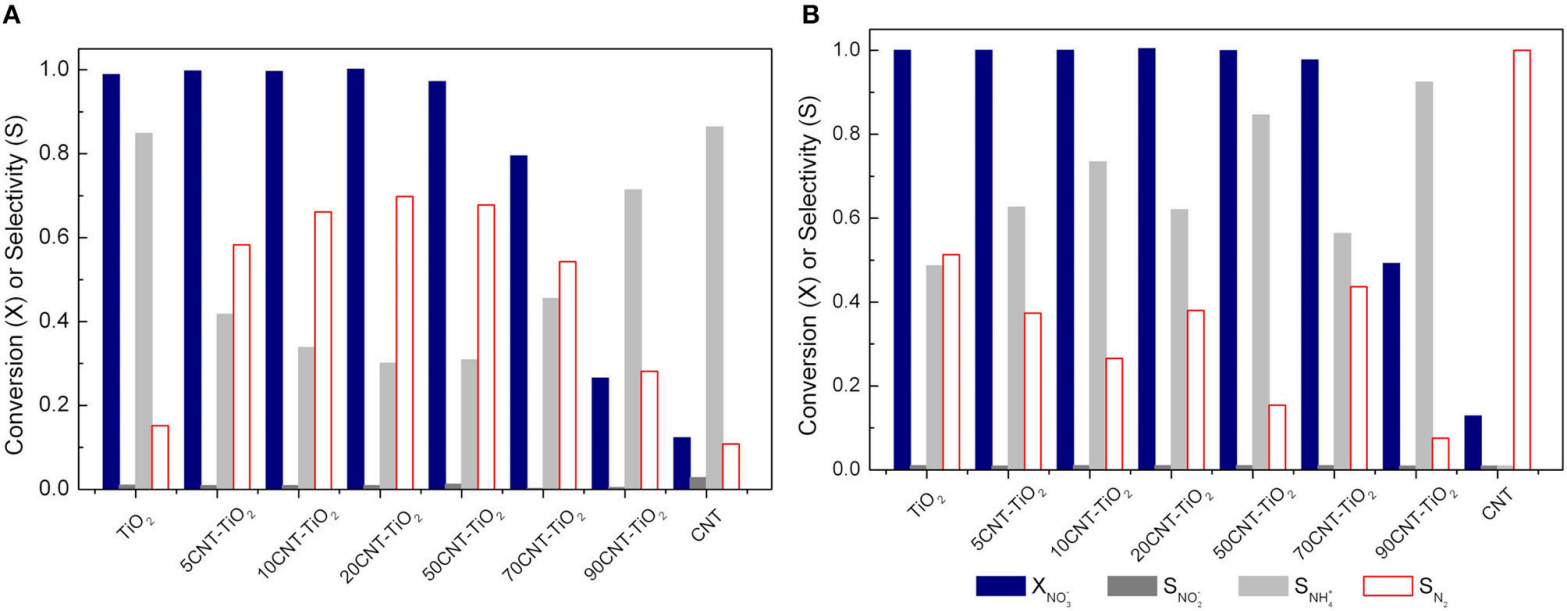
Nitrate conversion ($X_{NO_{3}{}^{-}}$) and nitrite, ammonium and nitrogen selectivity ($S_{{\mathrm{NO}}_{2}{}^{-}},S_{{{\mathrm{NH}}_{4}}^{+}},S_{N_{2}}$) using Pd-Cu loaded TiO_2_ and CNT-TiO_2_ catalysts after 4 h of catalytic **(A)** and photocatalytic **(B)** reactions.

As described above, nitrate reduction over CNT-TiO_2_ hybrid materials behave very differently in dark conditions and under irradiation. Although the photo-assisted process provide a quicker depletion of ${{\text{NO}}_{3}}^{-}$, the selectivity toward N_2_ is greatly affected by the use of CNT-TiO_2_ hybrid materials as Pd-Cu supports (Figure 8).

In the case of the catalytic reduction process, an increase in N_2_ selectivity is observed with increasing CNT content up to 20 wt.%. A further increase in the amount of CNT led to a progressive decrease in the selectivity toward N_2_ formation (Figure 8A). On the other hand, the photocatalytic reduction of ${{\text{NO}}_{3}}^{-}$ using CNT-TiO_2_ catalysts appeared to be more selective for the reduction of nitrate into ammonia (Figure 8B).

It is well accepted that during the (dark) catalytic reduction over bimetallic catalysts, using hydrogen as reducing agent, ${{\text{NO}}_{3}}^{-}$ is converted into ${{\text{NO}}_{2}}^{-}$ according to a redox reaction on the promoter metal (Cu). The role of the noble metal is to activate hydrogen, reducing the promoter metal, completing the catalytic cycle (Epron et al., 2001; Soares et al., 2011b; Zhang et al., 2013), being also active for the ${{\text{NO}}_{2}}^{-}$ reduction.

In the case of the photocatalytic process using metal-loaded TiO_2_, the mechanism generally proposed is based on the role of metal nanoparticles as electron sinks. Since the Fermi levels of noble metals are lower than that of TiO_2_, the photo-excited electrons can be transferred from the conduction band of the semiconductor to the metal nanoparticles deposited on its surface, being then available for ${{\text{NO}}_{3}}^{-}$ reduction (Kominami et al., 2005; Anderson, 2012; Soares et al., 2014). Hole scavengers are generally used, acting as sacrificial electron donors, avoiding electron-hole recombination. Yet, in the present work, no scavengers were added to the reaction medium.

The role played by CNT in CNT-TiO_2_ hybrids has been discussed in previous works reporting the use of this type of materials as photocatalysts for environmental applications (Silva and Faria, 2010; Silva et al., 2015; Zeng et al., 2015; Yang and Park, 2017). CNT may act as adsorbent, as dispersing medium for TiO_2_ nanoparticles, it may span light absorption into the visible and may retard electron hole recombination. Although the surface area of the hybrid materials increased with CNT content, the first is not likely to be the most important effect, since no significant adsorption was observed whether the catalyst used. TEM images of CNT-TiO_2_ hybrids show that CNT promote the dispersion of TiO_2_ avoiding particle agglomeration (Figure 2). Moreover, the introduction of CNT increased the absorption of the resulting materials in the visible range, as shown by the UV-Vis spectra (Figure 5A), and lead to a decrease in the electron-hole recombination (Figure 5B).

**Figure 9:**
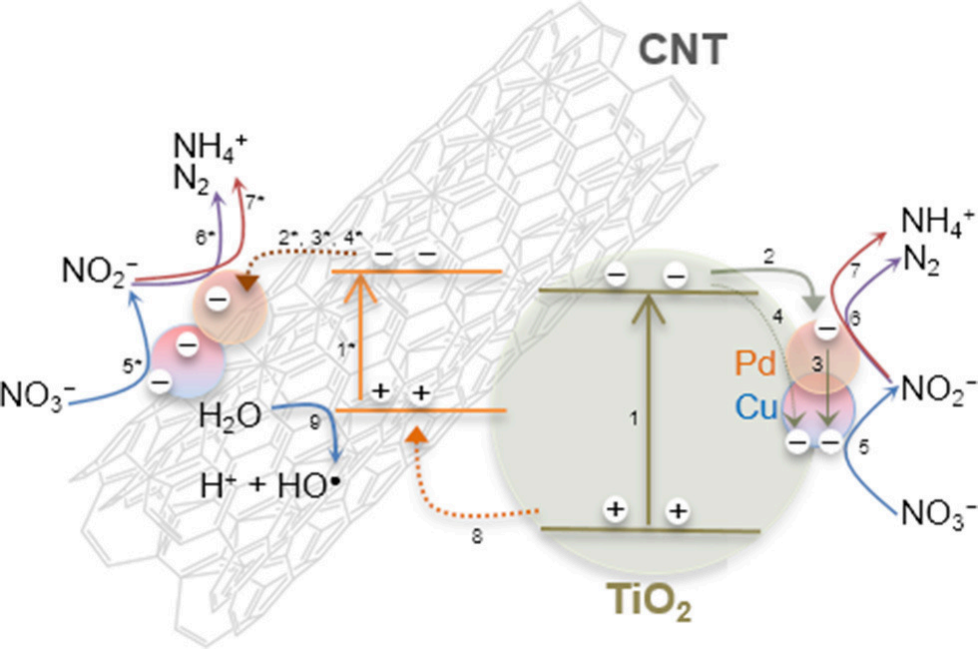
Schematic representation of the photocatalytic reduction of nitrate over Pd–Cu/CNT-TiO_2_ catalysts in the presence of H_2_ and CO_2_, under near UV to visible light irradiation.

Based in our findings, and considering the operation conditions used in this study, the following photocatalytic reaction mechanism is proposed. Since the irradiation source emits in the near UV to visible range, it is expected that TiO_2_ and CNT could be photoexcited simultaneously (Figure 9, steps 1 and 1^*^).

After charge separation, electrons are transferred to Pd and Cu nanoparticles that are supported over TiO_2_ and also over CNTs (Figure 9, steps 2–4), as observed by TEM (Figure 2). Photogenerated electrons may reduce both nitrate and nitrite adsorbed on Cu and Pd, respectively (Figure 9, steps 5–7). On the other hand, positively charged holes may migrate from TiO_2_ to the CNT phase, where, in the absence of sacrificial electron donors, water can be oxidized to H^+^ and HO^∙^ (Figure 9, steps 8 and 9). Hydroxyl radicals may indirectly re-oxidize byproducts to ${{\text{NO}}_{3}}^{-}$ (Tugaoen et al., 2017), while ${\text{CO}}_{2}{}^{∙-}$, which can be generated from the reduction of CO_2_ (used as pH buffer) by available electrons, may play a role as reducing mediator (Zhang et al., 2005; Sá et al., 2009). The higher selectivity toward ${\text{NH}}_{4}{}^{+}$ production obtained using CNT-TiO_2_ catalysts when irradiated may be rationalized by the excess of H^+^ in the reaction medium, resulting from step 9 in Figure 9. Yet, no direct correlation between the CNT load in the hybrid materials and the selectivity toward ${\text{NH}}_{4}{}^{+}$ could be found (Figure 8B), which may derive from the complexity and simultaneity of the reactions involved in the mechanism of the photocatalytic process.

Although the photocatalytic process appears more advantageous in terms of kinetics of nitrate removal, the (dark) catalytic reactions using CNT-TiO_2_ hybrid materials revealed to be more selective toward N_2_ formation. Nevertheless, the possibility of using this type of materials may be envisioned as a cleaner route for the production of ammonia, comparing with the conventional fossil fuel based process (Yamauchi et al., 2011; Hirakawa et al., 2017).

## Conclusion

TiO_2_ and CNT-TiO_2_ loaded with 1%Pd−1%Cu show high catalytic activity in the dark and under near UV to visible light irradiation, in the presence of H_2_ and CO_2_. The presence of light promotes faster ${{\text{NO}}_{3}}^{-}$ conversion, due to the higher availability of reducing species. Carbon nanotubes induce to a positive effect in the selectivity of the catalytic reduction process toward N_2_ formation. In the case of the photocatalytic process, the hybrid materials lead to an increase in the yield of the reduction of ${{\text{NO}}_{3}}^{-}$ to ${\text{NH}}_{4}{}^{+}$, due to the high availability of H^+^. The efficiency of the hybrid materials depends on the CNT load, the best performing material being that composed by 20 wt.% of carbon nanotubes. In the case of the dark catalytic process, the synergic effect observed by the introduction of CNT in the TiO_2_ matrix is mainly ascribed to the action of the carbon phase as dispersing medium to metal oxide particles, while under irradiation, CNT produce an increase in the efficiency of charge separation and mobility in the composite material.

## Author Contributions

CS and OS conceived the research work, prepared and characterized the catalysts, performed the activity tests and drafted the manuscript. JÓ, MP, and JF provided the means for the realization of this work and contributed to the interpretation of the experimental results. All authors read and approved the final manuscript.

### Conflict of Interest Statement

The authors declare that the research was conducted in the absence of any commercial or financial relationships that could be construed as a potential conflict of interest.

## Footnotes

^1^EU Water Framework Directive (2000). Available online at: http://ec.europa.eu/environment/water/water-framework/index_en.html (Accessed 2018).
